# Agent-specific DNA repair kinetic in 3T3 cells: a comparative study using the in vitro comet assay

**DOI:** 10.1093/toxres/tfaf180

**Published:** 2025-12-14

**Authors:** Seda İpek Tekneci, Aylin Üstündağ, Yalçın Duydu

**Affiliations:** Department of Pharmaceutical Toxicology, Faculty of Pharmacy, Ankara University, Ankara 06560, Türkiye; Graduate School of Health Sciences, Ankara University, Ankara 06110, Türkiye; Department of Pharmaceutical Toxicology, Faculty of Pharmacy, Ankara University, Ankara 06560, Türkiye; Department of Pharmaceutical Toxicology, Faculty of Pharmacy, Ankara University, Ankara 06560, Türkiye

**Keywords:** comet assay, DNA repair kinetics, genotoxicity, standardization, 3T3 cell line, positive controls, alkylating agents, oxidative DNA damage

## Abstract

Alkaline comet assay is a widely used technique for assessing DNA damage and repair at the single-cell level. However, the lack of standardized protocols, particularly for *in vitro* applications, makes it difficult to reproduce and compare the results from different laboratories. This study aimed to evaluate time-dependent DNA repair kinetics in 3T3 cells following exposure to six commonly used genotoxic agents, hydrogen peroxide (H_2_O_2_), potassium bromate (KBrO_3_), ethyl methanesulfonate (EMS), methyl methanesulfonate (MMS), N-ethyl-N-nitrosourea (ENU), and etoposide (EP). The cells were subjected to concentrations and exposure durations previously determined to induce maximum DNA damage. DNA repair was subsequently assessed at various intervals over a 24-h duration utilizing the comet assay. Each agent displayed a unique repair profile based on the type of DNA damage generated and cellular repair mechanisms involved. H_2_O_2_ and EMS induced rapid repair, whereas ENU- and EP-induced lesions were more persistent. These findings underscore the critical role of time as a variable in comet assay-based DNA repair assessments, contributing valuable reference data to support the standardization and validation of this assay for genotoxicity testing.

**Study highlights**

- The comet assay was used to assess DNA repair kinetics in 3T3 cells.
- Six genotoxic agents were tested: H_2_O_2_, KBrO_3_, EMS, MMS, ENU, and etoposide.
- Each agent displayed a unique repair profile based on the DNA damage type.
- H_2_O_2_ and EMS induced rapid repair, while ENU and etoposide lesions were persistent.
- Time is a critical variable in comet assay-based DNA repair assessment.
- The findings provide reference data to support comet assay standardization efforts.
- Considering agent-specific repair kinetics is crucial when interpreting comet assay results.

## Introduction

DNA is highly sensitive to both endogenous and exogenous agents.^1–3^ As a result of exposure to agents that cause DNA damage, various structural disorders such as base oxidation, alkylation, strand breaks, mismatched bases, DNA adductions, and intra-strand or inter-strand cross-links can occur.^3^ When the damage cannot be repaired, the cell cycle, gene expression, and genome integrity are negatively affected, leading to irreversible mutations and serious diseases such as cancer.^1^^,^^3^^,^^4^ However, to prevent permanent genome changes, cells have DNA repair mechanisms specific to the type of damage, such as base excision repair (BER), nucleotide excision repair (NER), mismatch repair (MMR), homologous recombination, and non-homologous end joining, which play critical roles in preserving genetic information and maintaining cellular stability.^3–6^ The capacity of repair mechanisms varies according to the type and degree of damage as well as the cell type.^1^^,^^3^^,^^7^ Therefore, assessment of DNA damage and repair is of great importance in understanding the ability of cells to maintain genomic integrity.

One of the most widely used methods for assessing DNA damage and repair capacity at the single-cell level is the comet assay.^3–5^^,^^8^ The comet assay has become a frequently used method in genotoxicity studies because of its ease of application, ability to work with small amounts of sample, and high sensitivity.^9^ This test, which was first developed in the 1980s to detect DNA strand breaks, was made more comprehensive in the following years with modifications to detect different DNA lesions, such as oxidized bases.^10^ The comet assay, which is used in both *in vivo* and *in vitro* genotoxicity studies, is an important tool for the investigation of DNA damage and repair mechanisms.^8^

In genotoxicity studies, the DNA repair capacity can be assessed with various modifications using the comet test, which allows the assessment of DNA damage. One of the methods of assessing DNA repair capacity using the comet test is based on the decrease in DNA damage in cells over time.^7^ To correctly interpret both DNA damage and repair capacity using the comet test, the test procedures must be carried out carefully, and the reliability of the results must be ensured. The most important problem encountered, especially in the *in vitro* comet test, is the use of different protocols between laboratories, and the lack of sufficient detailed information regarding the test protocols used.^2^^,^^11^^,^^12^ This situation raises questions regarding the reliability of the results obtained.^6^ Standardization and validation of the method are of great importance in terms of increasing the reliability of the results obtained with the comet test and enabling inter-laboratory comparability.^3^^,^^8^^,^^12^^,^^13^ The European Comet Test Validation Group (ECVAG) and European Standards Committee are conducting international standardization studies to obtain more consistent and reproducible results in the measurement of DNA damage.^2^ In particular, the fact that the *in vitro* comet test does not have a standard protocol that is internationally accepted in toxicity test guidelines reveals the importance of conducting studies in this field. In this context, studies carried out to minimize inter-laboratory protocol differences for the *in vitro* comet test will increase the scientific reliability of the test.

DNA damage in the comet test was affected by the DNA repair processes of the cells. In this respect, determining the time points when DNA damage reaches its maximum and repair begins is important for evaluating comet test results.^7^ In this study, we aimed to determine DNA repair in the 3T3 (Swiss albino) cell line with different positive controls known to cause different types of DNA damage using the *in vitro* alkaline comet test method. In our previous study, we determined the exposure time at which the same positive controls caused maximum DNA damage in the same cell line. In addition, in our previous study, cell viability during the period when maximum DNA damage occurred was evaluated. In this study, we aimed to provide data that will contribute to both the accurate evaluation of interlaboratory test results and standardization of the test method by determining the DNA repair capacity of the 3T3 cell line using the standard alkaline comet test method.

## Materials and methods

### Cell culture

The 3T3 (ATCC® CCL-92, Germany) Swiss albino cell line was used in this study. These cells were chosen because they are a widely used and well-characterized fibroblast model, making them suitable for genotoxicity and DNA repair studies. The selected cell line was grown in a Dulbecco’s Modified Eagle Medium (high glucose, DMEM) containing 10% Fetal bovine serum (FBS), 1% penicillin/streptomycin and 5% CO_2_ at 37 °C. When the cells reached 80% confluency, they were detached from the surface with 0.25% trypsin/EDTA, and the cells were seeded in fresh medium for a certain period of time to be ready for the comet test.

On the first day of DNA repair with the comet test, cells were seeded in 6-well plates as 200,000 cells. On the day of the experiment, the cells were exposed to the chemicals to be tested for 30 min for hydrogen peroxide (H_2_O_2_), etoposide (EP), N-ethyl-N-nitrosourea (ENU), and potassium bromate (KBrO_3_); 60 min for ethyl methanesulfonate (EMS) and methyl methanesulfonate (MMS) and concentrations (H_2_O_2_ 50 μM; MMS 500 μM; EP 10 μM; EMS 2 mM; ENU 2 mM; KBrO_3_ 2.5 mM) at which maximum DNA damage occurred, as determined in our previous study. In addition, cell viability data for the determined time and concentration values were included in our previous study.^14^

### Reagents

H_2_O_2_, MMS, EMS, ENU, KBrO_3_, normal- and low-melting-point agarose, 0.25% trypsin–EDTA, penicillin/streptomycin (Pen/Strep), dimethyl sulfoxide (DMSO), and ethidium bromide were purchased from Sigma-Aldrich, Germany. EP was purchased from Cayman Chemical (USA). DMEM and trypsin were purchased from Sartorius, Israel. FBS (heat-inactivated) was obtained from Biological Industries, Israel. Hydrochloric acid (HCl), sodium hydroxide (NaOH), Tris, and Triton X-100 were supplied by Merck, Germany, whereas disodium ethylenediaminetetraacetic acid (Na_2_EDTA), sodium chloride, and sodium lauryl sarcosinate were purchased from Amresco (USA).

### DNA repair

Time-dependent DNA repair in cells was performed using the standard *in vitro* alkaline comet test procedure.^15^ The medium of the cells exposed to the determined concentrations was replaced with fresh medium at the end of the periods that caused maximum DNA damage. One milliliter of 3T3 cell suspension was used to perform the alkaline comet test at 15, 30, and 45 min and 1, 2, 4, 6, 8, 16, and 24 h after the addition of fresh medium. The cell suspension was centrifuged at 1000 rpm for 5 min, the supernatant was discarded, and the cells were suspended in PBS. The cell suspension (50 μL) was mixed with 100 μL of low-melting-point agarose (0.5%) and spread on slides previously coated with normal-melting-point agarose. The prepared slides were left to freeze on cold metal surfaces. The slides were then kept in the dark for 1 h in freshly prepared lysing solution (10 mM Tris, 2.5 M NaCl, 100 mM Na_2_EDTA, 1% sodium sarcosinate, 1% Triton-X 100, 10% DMSO at pH = 10.0) at +4^0^C. At the end of the lysing period, the slides were washed once with distilled water and placed in cold electrophoresis solution (pH > 13). After waiting for 20 min without applying current, electrophoresis was performed for 20 min at 300 mA and 0.7 V/cm. Following electrophoresis, the slides were exposed to distilled water for 5 min, neutralization solution (0.4 M Tris at pH = 7.5) for 15 min, and distilled water for 5 min. For subsequent imaging, slides were fixed with 50%, 70%, and 100% alcohol. Imaging and scoring were performed using the COMET Assay IV Software program (Instem, UK) under a Leica DM fluorescent microscope (Germany), and 100 cells were randomly counted after staining with 60 μL (20 μg/mL) ethidium bromide solution. For each reagent, duplicate slides were prepared and evaluated in a blinded manner. Time-dependent DNA repair was determined based on the decrease in the mean tail density.

### Statistical analysis

Statistical analysis was conducted using the SPSS software (Version 23.0, SPSS Inc., USA). Results are presented as the mean ± standard error of the mean (SEM). One-way analysis of variance (ANOVA) was performed to examine all chemical groups and exposure durations. Post hoc analysis, specifically utilizing Fisher’s least significant difference test (LSD), was conducted for intergroup comparisons. Statistical significance was set at *P* < 0.05.

## Results

In our previous study, the concentrations and exposure durations that resulted in maximum DNA damage were determined for H_2_O_2_, KBrO_3_, MMS, EMS, ENU, and EP in a 3T3 cell line. The cell viability of the samples was also evaluated at the determined concentrations and durations. As illustrated in Table 1, the concentrations and exposure durations employed for the assessment of DNA repair were derived from the dataset obtained in our previous study.^14^

**Table 1:** Data of 6 different positive controls for the concentration-exposure duration-mean % DNA in tail determined in the previous study.^14^

Reagent	Concentration	Exposure Duration	DNA in Tail (%Mean)
H_2_O_2_	50 μM	30 min.	46.35
KBrO_3_	2.5 mM	30 min.	7.38
EMS	2 mM	1 h.	10.54
MMS	500 μM	1 h.	27.04
ENU	2 mM	30 min.	16.48
EP	10 μM	30 min.	33.85

### DNA repair over time

To evaluate DNA repair over time, 3 T3 cells were treated with each chemical at the concentration and exposure duration that caused the maximum DNA damage using the alkaline comet assay. Following the exposure, the culture medium containing the chemicals was replaced with fresh medium.

**Fig. 1:**
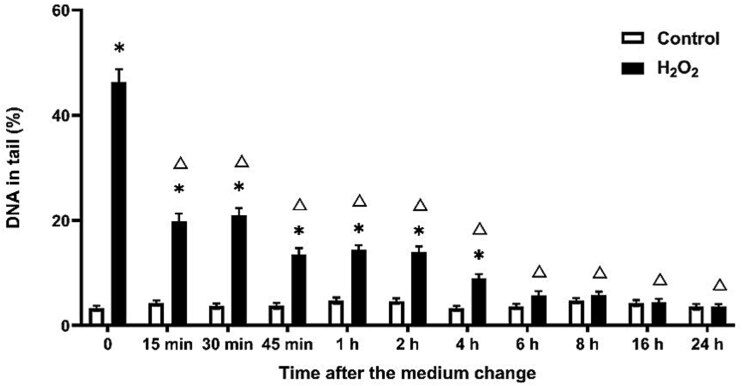
DNA damage reduction after H_2_O_2_ treatment over time in the comet assay. Exposure duration was 0.5 h and DNA damage was measured after medium change. ^*^P ≤ 0.05 vs. control at the same time point and Δ ≤ 0.05 vs. 50 μM H_2_O_2_ at 0 h.

As demonstrated in Fig. 1, a substantial decline in H_2_O_2_-induced DNA damage was observed starting at 15 min, and there was no significant difference between the control group after 6 h.

**Fig. 2:**
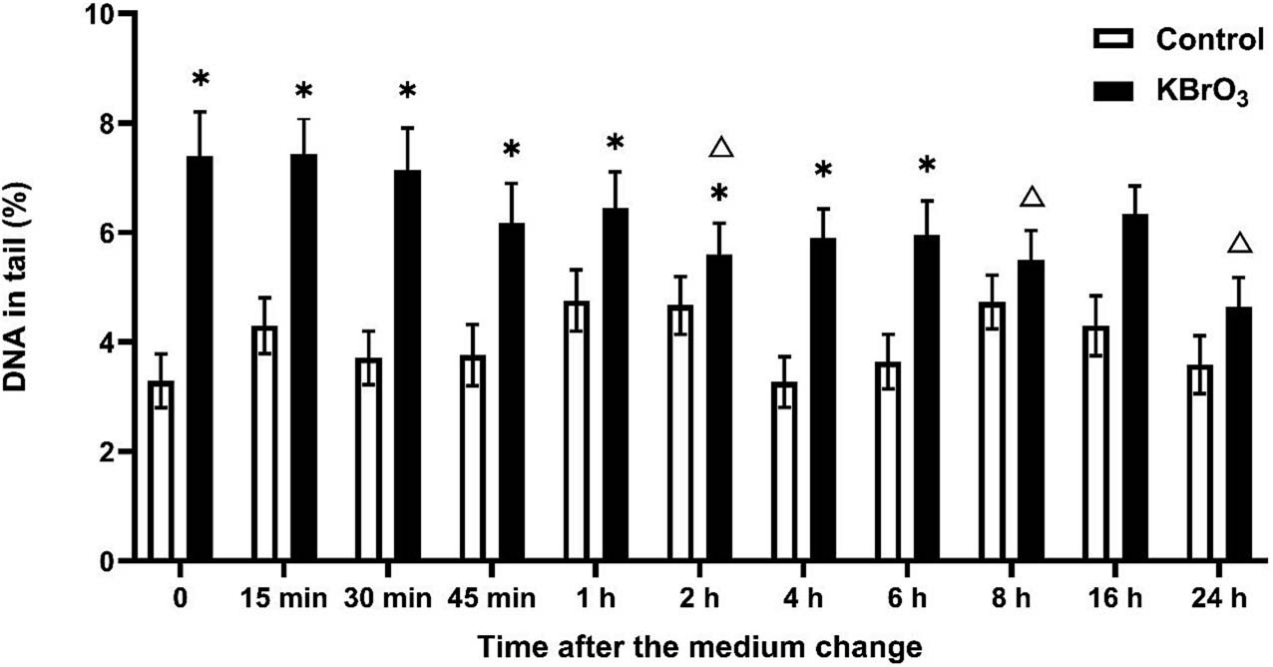
DNA damage reduction after KBrO_3_ treatment over time in the comet assay. Exposure duration was 0.5 h and DNA damage was measured after medium change. ^*^P ≤ 0.05 vs. control at the same time point and Δ ≤ 0.05 vs. 2,500 μM KBrO_3_ at 0 h.

Figure 2 shows that a significant decrease in KBrO_3_-induced DNA damage started in the 2nd hour. No statistically significant differences were observed in comparison with the control group, starting from the 8th hour.

**Fig. 3:**
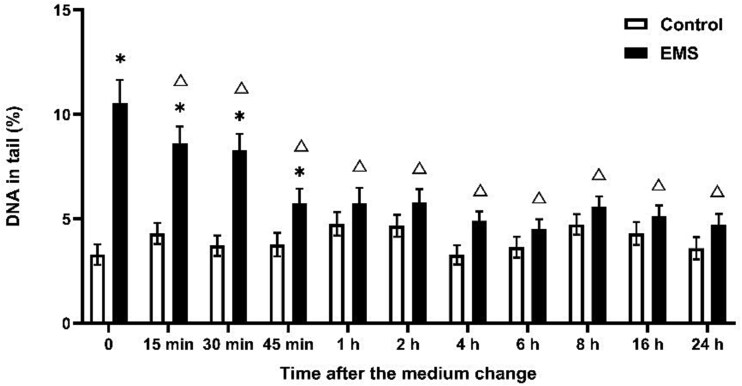
DNA damage reduction after EMS treatment over time in the comet assay. Exposure duration was 1 h and DNA damage was measured after medium change. ^*^P ≤ 0.05 vs. control at the same time point and Δ ≤ 0.05 vs. 2 mM EMS at 0 h.

**Fig. 4:**
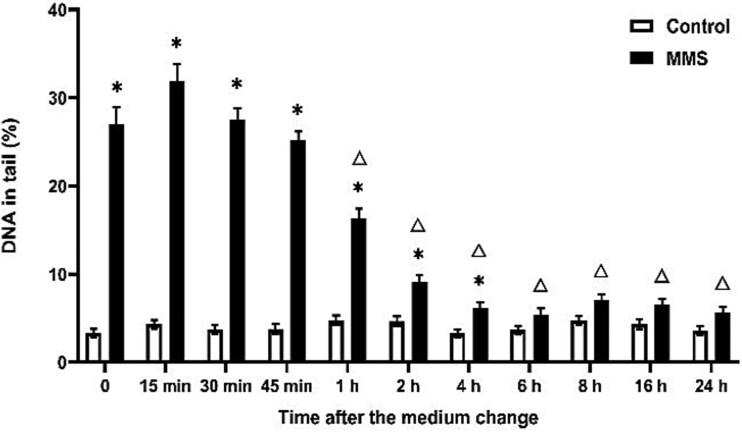
DNA damage reduction after MMS treatment over time in the comet assay. Exposure duration was 1 h and DNA damage was measured after medium change. ^*^P ≤ 0.05 vs. control at the same time point and Δ ≤ 0.05 vs. 500 μM MMS at 0 h.

**Fig. 5:**
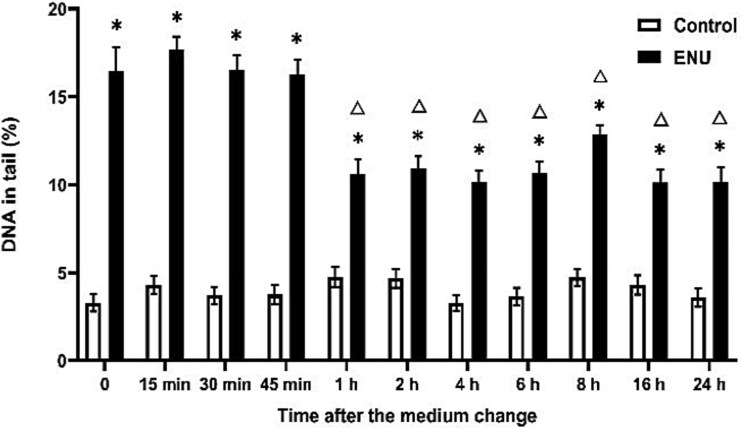
DNA damage reduction after ENU treatment over time in the comet assay. Exposure duration was 0.5 h and DNA damage was measured after medium change. ^*^P ≤ 0.05 vs. control at the same time point and Δ ≤ 0.05 vs. 2 mM ENU at 0 h.

EMS, MMS, and ENU exhibit different DNA repair profiles. The decrease in DNA damage induced by EMS began at 15 min, whereas for MMS and ENU, this decrease was observed at 1 hour (Figs 3–5). EMS- and MMS-induced DNA damage approached the control group levels at 1 h and 6 h, respectively, and the significant difference between them disappeared (Figs 3 and 4). However, ENU-induced DNA damage was significantly different from that in the control group on the next day (Fig. 5).

**Fig. 6:**
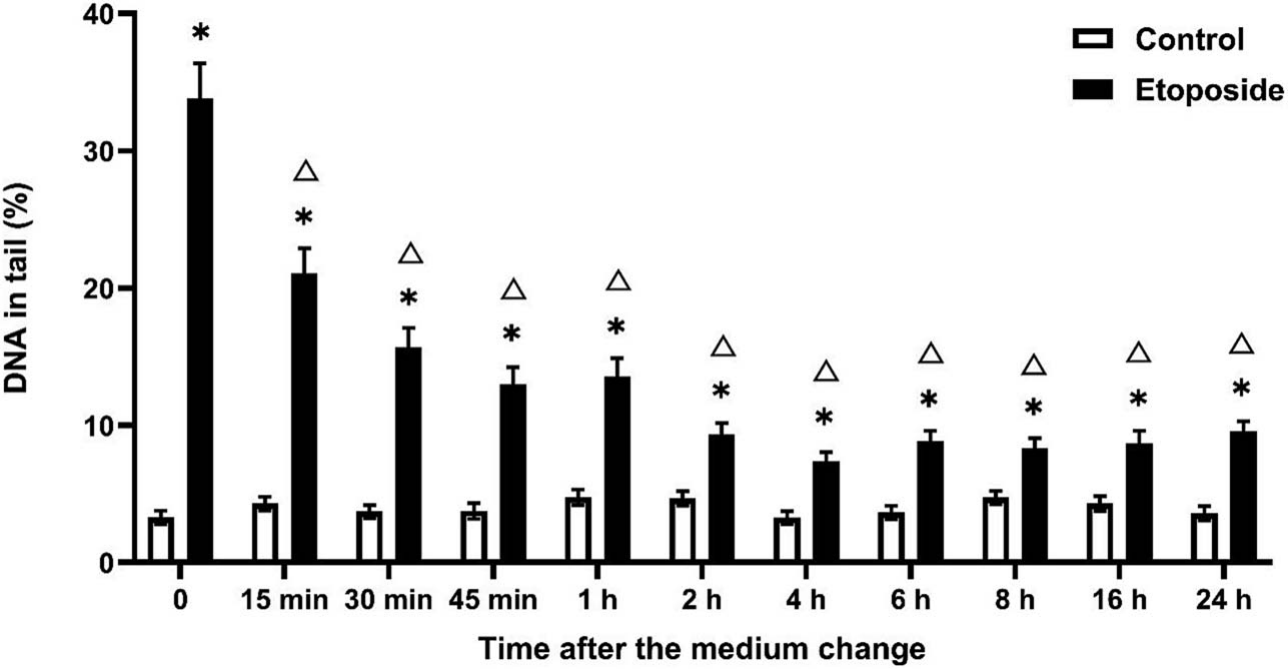
DNA damage reduction after EP treatment over time in the comet assay. Exposure duration was 0.5 h and DNA damage was measured after medium change. ^*^P ≤ 0.05 vs. control at the same time point and Δ ≤ 0.05 vs. 10 μM etoposide at 0 h.

Figure 6 shows that EP-induced DNA damage started to decrease after 15 min. However, it was determined that DNA damage did not reach the control level on the day after the medium change.

Finally, Table 2 provides a comparative summary of the DNA repair profiles of different types of DNA-damaging agents in 3 T3 cells.

**Table 2:** Summary of the types of DNA damage induced by the tested agents, their mechanisms of action, and the observed onset of repair in the 3T3 cell line.

Reagent	Type of DNA damage	Mechanism of action	Onset of repair *(depending on our results)*	Reference
H_2_O_2_	Oxidative base modifications, single-strand breaks	Generation of hydroxyl radicals (•OH) via Fenton/Haber-Weiss reactions	From 15 min.	*21,22*
KBrO_3_	Oxidative base lesions (mainly 8-oxoG)	Reduction by cellular thiols forming bromine radicals and oxides	From 2 h.	*28,29*
EMS	N7-guanine and N3-adenine alkylation	Direct alkylation of nucleophilic DNA sites	From 15 min.	*30,31*
MMS	N7-guanine and N3-adenine alkylation	Direct alkylation of nucleophilic DNA sites	From 1 h.	*30,31*
ENU	O6-guanine ethylation	Direct alkylation of nucleophilic DNA sites	From 1 h.	*30,31,32*
EP	Double-strand breaks (DSBs)	Inhibition of topoisomerase IIα	From 15 min.	*36,37*

## Discussion

Precise evaluation of DNA damage and repair is essential for understanding the ability of cells to preserve their genomic integrity. The comet assay is a widely used genotoxicity testing tool to evaluate DNA damage and repair at the single-cell level. Cellular DNA repair ability can be assessed using several versions of the comet assay, such as the reduction in DNA damage over time, utilizing the aphidicolin block reagent assay, and including cellular extracts that contain repair enzymes.^4^^,^^16–20^ The first two approaches can achieve repair outcomes shortly after the onset of DNA damage. The utilization of cellular extracts containing repair enzymes requires a substantial number of cells.^1^

The objective of this study was to examine the time-dependent repair of DNA damage in 3 T3 cells induced by various positive control agents, using an *in vitro* alkaline comet assay. The results revealed that the DNA damage induced by each agent had different DNA repair profiles depending on the cellular repair capacity.

H_2_O_2_, a comparatively stable reactive oxygen species, induces DNA damage primarily by generating highly reactive hydroxyl radicals (•OH) via the Fenton and Haber-Weiss processes involving transition metals such as Fe^2+^. •OH causes considerable oxidative damage to DNA, leading to base alterations, abasic sites, intrastrand crosslinks, strand breakage, and DNA-protein crosslinks.^21^^,^^22^ DNA damage caused by H_2_O_2_ is primarily repaired through base excision repair (BER), but the nucleotide excision process (NER) may play a minor role.^7^^,^^22^ The response of cells to H_2_O_2_-induced damage varies depending on the cell type, physiological state, concentration, and time of exposure.^23^^,^^24^ It was reported that HeLa cells exposed to H_2_O_2_ for 30 min on ice rapidly repaired the DNA damage caused by hydrogen peroxide within 1 h.^25^ In a previous study, it was shown that DNA damage in HepG2 cells exposed to H_2_O_2_ for 5 min, 30 min, 40 min, 1 h, and 24 h did not differ from the control group after 24 h.^26^ In a study showing the difference between DNA repair capacity according to cell type, H_2_O_2_-induced DNA damage repair was faster in TK6 cells than in human lymphocyte cells.^27^ In addition, the growth stage of cells affects the activities of glutathione reductase, glutathione peroxidase, and catalase enzymes, which are responsible for the detoxification of H_2_O_2_.^23^^,^^25^ While these antioxidant enzymes are essential for mitigating the initial ROS burden, the resulting cellular response is not *solely* dictated by their activity levels. The efficiency of the DNA repair pathways, which are responsible for removing the oxidative DNA lesions that inevitably form, is also a critical factor. Indeed, evidence suggests that the overall DNA repair capacity can be a dominant determinant in explaining the differences in cellular sensitivity and response to oxidative damage.^25^ In our study, the rapid reduction of oxidative damage caused by H_2_O_2_ in the 3T3 cell line indicated that oxidative base modifications were effectively repaired by the base excision repair process. However, even if the initial repair process started rapidly, the decrease in damage approached that of the control group after the 6th hour, suggesting that the proteins involved in the repair processes may be affected by cellular expression levels or the stages of the cell cycle. Similar to H_2_O_2_, KBrO_3_ is a well-recognized oxidative agent that primarily induces the formation of 7,8-dihydro-8-oxo-guanine (8-oxoG) in DNA.^28^ Unlike H_2_O_2_, it does not require transition metals or enzymes for its damage mechanism. It is thought to cause DNA damage by the formation of reactive intermediates containing bromine radicals (Br•) or oxides (BrO•, BrO₂• vb.) by the reduction of bromate by thiols such as cellular glutathione (GSH).^28^^,^^29^ The effects of both oxidant agents on some DNA repair genes such as BRCA1, APEX1, and MUTYH have been shown to be different.^29^ This may explain the delayed onset of damage repair caused by potassium bromate in the cell line used in our study, compared to H_2_O_2_.

MMS, EMS and ENU are in the class of the alkylating agents that bind alkyl groups to nucleophilic sites in DNA, resulting in structural modifications that may disrupt its functionality.^30^^,^^31^ MMS and EMS primarily induce N7-G adducts, while ENU potently induces O adducts in DNA.^30^^,^^32^ Alkylation-induced base modifications in DNA are reversed through a BER, NER, mismatch repair (MMR), and direct reversal mechanism mediated by O6-methylguanine-DNA.^33^^,^^34^ The repair kinetics of DNA damage caused by different alkylating agents used in our study were different in the 3T3 cell line. Damage repair caused by EMS was faster than that caused by MMS and ENU, and approached the control group in a shorter time. Even though the damage caused by ENU started to decrease from the 1st hour, the significant difference from the control group continued even at the 24th hour. ENU adducts at the O6-guanine position in DNA, and this is a very stable structure. If the O6-methylguanine-DNA methyltransferase (MGMT) enzyme involved in repair is not sufficiently present in the cell, the repair process may be prolonged. The cellular response to MMS may differ according to cell line type. The damage induced by MMS in TK6 cells started to decrease from the 3rd hour and approached that of the control group at the 20th hour.^7^ In fresh human blood samples, half of the DNA damage was repaired within 1 hour.^35^ Viau et al. (2009) found that half of MMS-induced damage in V79 cells was repaired between 2 and 4 hours.

Etoposide is a topoisomerase IIα inhibitor that acts as an anticancer agent. It effectively induced DNA double-strand breaks, leading to cell cycle arrest and cell death. Damage is predominantly repaired via non-homologous end joining (NHEJ) and homologous recombination (HR) processes, depending on the cell cycle phase.^36^^,^^37^ If EP-induced double-strand breaks occur during the G1 phase of the cell cycle, then the NHEJ pathway is primarily involved in repair. In contrast, if damage occurs during the G2 or S phases, the HR pathway becomes active. The HR pathway provides more accurate repair of double-strand breaks than the NHEJ pathway. However, the NHEJ process is typically regarded as the primary mechanism for repairing etoposide-induced DNA damage generated by etoposide. EP is more effective in cancer cells with a high proliferation rate because of the increased DNA replication and transcription processes.^38^ Fibroblast-derived 3T3 cells have limited proliferation capacity. These findings indicate that the NHEJ pathway may have been the primary factor in the repair process, as evidenced by the rapid reduction in etoposide-induced damage. However, a significant difference was observed between the EP-treated and control groups even after 24 hours. This suggests that in a cell line with limited proliferation capacity, the HR pathway may have shown limited activity, other than the NHEJ pathway, in the repair of double-strand breaks. In addition, the repair of double-strand breaks requires a more complex and lengthy process compared to other types of DNA damage. This may explain the incomplete repair of the existing damage.

This study aimed to evaluate different agents capable of inducing various types of DNA damage. Consequently, the repair kinetic profiles of different damage types were determined using a comet assay. The 3T3 cell line preferred in this study increased the reliability of the study in terms of its use in genotoxicity tests, which is frequently preferred in the literature. However, when the limitations of our study are evaluated, the alkaline comet assay was insufficient to evaluate the repair of specific DNA adducts. Therefore, it is important that future studies be supported by more advanced techniques to determine DNA adducts using methods such as HPLC-MS/MS and to elucidate repair pathways.

The present study emphasizes the necessity of considering agent-specific kinetics when interpreting DNA repair using an *in vitro* comet assay, thereby contributing to ongoing efforts for assay standardization. The findings show that the time factor is a critical variable for each agent in the evaluation of cellular repair capacity. The comet assay is a valuable method not only for the measurement of damage at the cellular level but also for the evaluation of repair kinetics. However, while this study provides valuable insights into the time-dependent and agent-specific nature of DNA repair kinetics using the alkaline comet assay, it has certain limitations inherent in the experimental design. The reliance solely on the quantification of % tail DNA offers a phenomenological view of DNA repair rather than a deep mechanistic understanding. For further studies, additional molecular endpoints- DNA repair genes and specific DNA lesions (e.g. 8-oxoG), activity or protein levels of specific repair enzymes, the status of antioxidant defense markers- would undoubtedly strengthen the interpretation of our findings. These approaches would provide a more comprehensive and mechanistic picture, clarifying how these distinct repair profiles are regulated at the molecular level. Our current findings establish a critical baseline for these future, more detailed investigations.

## Conclusion

In this study, we systematically investigated the time-dependent DNA repair kinetics of 3T3 fibroblast cells in response to six commonly used genotoxic agents using an *in vitro* alkaline comet assay. The DNA repair profile of each agent depends on the nature of the induced damage and the cellular repair mechanisms involved. Oxidative DNA lesions caused by H_2_O_2_ and KBrO_3_ were repaired comparatively quickly, whereas alkylating agents displayed variable repair timelines. ENU-induced lesions were the most persistent. EP-induced strand breaks were partially resolved, but not fully repaired, even after 24 h, suggesting the involvement of slower, more complex repair pathways.

These results emphasize the importance of considering agent-specific DNA repair kinetics when interpreting the genotoxicity data obtained from the comet assay. Our results also underscore the critical role of time as a variable for interpreting DNA damage and repair. By characterizing the repair kinetics of well-established positive controls under standardized conditions, this study provides valuable reference data that could support the harmonization and validation of the *in vitro* comet assay for regulatory toxicology and mechanistic research.

## Data Availability

All data generated or analyzed during this study are included in this published article. Additional inquiries can be directed to the corresponding author.
